# Development and fine-tuning of a scale down model for process characterization studies of a monoclonal antibody upstream production process

**DOI:** 10.1186/1753-6561-5-S8-P70

**Published:** 2011-11-22

**Authors:** Mareike Harmsen, Jimmy Stofferis, Laetitia Malphettes

**Affiliations:** 1 Cell Culture Process Development Group, Biological Process Development, UCB Pharma S.A, Braine-l’Alleud, 1420, Belgium

## Background

It has always been an objective of process development and more recently it has also become a regulatory expectation to build robustness into and demonstrate proper control of a manufacturing process, thus ensuring that the biological product meets consistently its quality attributes and specifications. This is achieved mainly through systematic process development and understanding. Once a process is locked and ahead of consistency runs at the intended commercial scale, process characterization studies (PCS hereafter) further contribute to the demonstration of process robustness and the justification of process control ranges. These studies characterize the relationship between process parameters and process performance as well as product quality attributes. For practical reasons PCS are performed in a scale down model (SDM hereafter) of the manufacturing process studied. Therefore, it is essential to establish a SDM that is representative of the commercial scale.

Here we describe a road map for developing a scale down model of a cell culture process for recombinant protein production. The cell culture process modeled was a 12,000 L scale fed batch process producing a monoclonal antibody.

## Methods

In a first step, the SDM was defined such that volume- dependent parameters (medium and feeds volumes, working volumes) were scaled down linearly and volume-independent ones (temperature, dissolved oxygen, pH, cell age) were kept at the same set-points as in the commercial scale bioreactor. Some cell culture process parameters such as agitation and aeration are difficult to scale down linearly due to bioreactor and sparger geometry and differences in controller systems. Hence determination of these parameters was first done by engineering scale down considerations. These considerations determined a theoretical small scale model used for proof of concept experiments. Process performance in initial scale down runs was then compared to commercial scale production in terms of cell growth, key metabolites, product accumulation.

Two main deliverables were defined for the SDM**:**

1. The model should exhibit process performance comparable to the commercial scale process. This was necessary to study the effects of input parameter variations during PCS.

A way of assessing process performance is by monitoring in-process parameters during the SDM runs and comparing them to commercial scale batch data. Here the following evaluation parameters were chosen:

• Viable cell density and viabilities

• Metabolism indicators (namely off-line pH, dCO_2_, osmolality, glucose and lactate concentrations)

• Product titer

2. The model should deliver material with product quality attributes comparable to the material produced at the corresponding manufacturing scale process step (post cell harvest). This was necessary to study the effects of parameter changes on upstream product quality.

Here the evaluation parameters were based on selected product quality specifications for the commercial scale batches. Namely:

• Oligosaccharide profiles

• Monomer/aggregate/fragment proportions

• Monomer,- Heavy-and Light chain patterns

• Acidic peak group species proportions

## Results

Proof of concept bioreactor runs using the theoretical SDM showed good comparability between the model and the commercial scale regarding process performance; however it was observed that the dissolved CO_2_ values were lower than in commercial scale. We consider this to be a consequence of geometrical bioreactor differences. The liquid surface-to-volume ratio is significantly higher in small scale than it is in large scale. Furthermore in small scale bioreactors a higher maximum volumetric gas flow rate was employed to compensate for a reduced gas bubble residence time. These two main differences resulted in increased CO_2_ stripping.

An overlay flow containing 6 % CO_2_ was applied to the headspace of the 2 L vessels to reduce the stripping (fine –tuning).

After establishing the CO_2_ overlay as part of the aeration strategy, three SDM verification runs were carried out. Cell growth, cell metabolism and Mab titers were monitored throughout the cultures. Lab scale data resulting from daily sampling was mostly within the average ± 3 x standard deviation (SD) of the commercial scale data and therefore comparable.

Scale down model harvest material was compared to harvest material from the commercial scale with regards to their product quality attributes. The clarified cell culture fluid from both scales was protein A purified in laboratory scale and product quality was assessed (Table 1).

**Table 1:** Product quality attributes of purified harvest material

Product quality attribute	Commercial scale reference sample	SDM verification run 1	SDM verification run 2	SDM verification run 3
Oligosaccharide mapping	Profile comparable to standard	Profile comparable to standard	Profile comparable to standard	Profile comparable to standard

Purity by size exclusion HPLC				

Monomer [%]	99	100	99	99

Aggregate [%]	1	0	0	1

Fragment [%]	0	0	1	0

Monomer pattern by capillary gel electrophoresis				

Monomer [%]	97	97	97	97

Light and heavy chain pattern by capillary gel electrophoresis				

Light chain + heavy chain [%]	99	99	98	99

Acidic species by cation exchange chromatography				

Acidic species [%]	30	30	28	32

## Conclusions

Good comparability regarding process performance of the production bioreactor between the SDM runs and large scale data was demonstrated. Growth data, product titer, pH, osmolality, glucose and lactate profiles all showed the same trends. The tested output parameters where mostly within ± 3 x SD of the average commercial scale data.

Product quality attributes of scale down samples were comparable to commercial scale product quality as shown by comparison with clarified cell culture fluid samples from commercial scale.

The verification results demonstrate that the systematic scale down approach applied in this work provided a laboratory scale model that was representative of the commercial scale process in terms of process performance and product quality of the generated material and was therefore suitable to be used in process characterization studies.

